# Dissecting trait heterogeneity: a comparison of three clustering methods applied to genotypic data

**DOI:** 10.1186/1471-2105-7-204

**Published:** 2006-04-12

**Authors:** Tricia A Thornton-Wells, Jason H Moore, Jonathan L Haines

**Affiliations:** 1 Neuroscience Graduate Program, Vanderbilt Brain Institute, Vanderbilt University Medical Center, Nashville, TN, USA; 2 Department of Biomedical Informatics, Vanderbilt University Medical Center, Nashville, TN, USA; 3 Center for Human Genetics Research, Vanderbilt University Medical Center, Nashville, TN, USA; 4 Computational Genetics Laboratory, Department of Genetics, Dartmouth Medical School, Lebanon, NH, USA; 5 Department of Molecular Physiology and Biophysics, Vanderbilt University Medical Center, Nashville, TN, USA

## Abstract

**Background:**

Trait heterogeneity, which exists when a trait has been defined with insufficient specificity such that it is actually two or more distinct traits, has been implicated as a confounding factor in traditional statistical genetics of complex human disease. In the absence of detailed phenotypic data collected consistently in combination with genetic data, unsupervised computational methodologies offer the potential for discovering underlying trait heterogeneity. The performance of three such methods – Bayesian Classification, Hypergraph-Based Clustering, and Fuzzy *k*-Modes Clustering – appropriate for categorical data were compared. Also tested was the ability of these methods to detect trait heterogeneity in the presence of locus heterogeneity and/or gene-gene interaction, which are two other complicating factors in discovering genetic models of complex human disease. To determine the efficacy of applying the Bayesian Classification method to real data, the reliability of its internal clustering metrics at finding good clusterings was evaluated using permutation testing.

**Results:**

Bayesian Classification outperformed the other two methods, with the exception that the Fuzzy *k*-Modes Clustering performed best on the most complex genetic model. Bayesian Classification achieved excellent recovery for 75% of the datasets simulated under the simplest genetic model, while it achieved moderate recovery for 56% of datasets with a sample size of 500 or more (across all simulated models) and for 86% of datasets with 10 or fewer nonfunctional loci (across all simulated models). Neither Hypergraph Clustering nor Fuzzy *k*-Modes Clustering achieved good or excellent cluster recovery for a majority of datasets even under a restricted set of conditions. When using the average log of class strength as the internal clustering metric, the false positive rate was controlled very well, at three percent or less for all three significance levels (0.01, 0.05, 0.10), and the false negative rate was acceptably low (18 percent) for the least stringent significance level of 0.10.

**Conclusion:**

Bayesian Classification shows promise as an unsupervised computational method for dissecting trait heterogeneity in genotypic data. Its control of false positive and false negative rates lends confidence to the validity of its results. Further investigation of how different parameter settings may improve the performance of Bayesian Classification, especially under more complex genetic models, is ongoing.

## Background

### Complex human genetic disease

**Figure 1:**
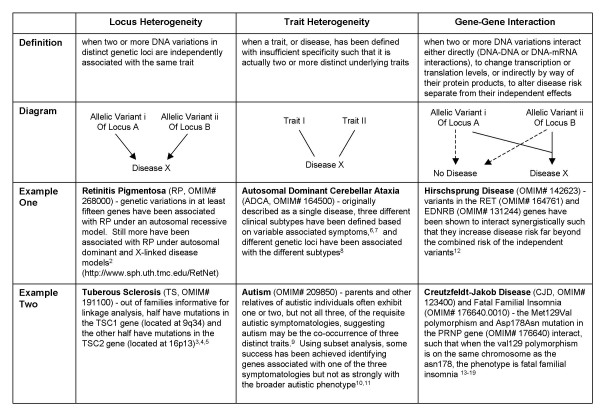
Factors Complicating Analysis of Complex Genetic Disease: Definitions, Diagrams and Examples

Molecular biologists and geneticists alike now acknowledge that the most common human diseases with a genetic component are likely to have very complex etiologies, involving such complicating factors as locus heterogeneity, trait heterogeneity, and gene-gene interactions (Figure 1; [1-19]). However, only a small fraction of the human genetics literature specifically reports on investigations of such complexity. Statistical geneticists continue primarily using traditional methodologies, such as linkage and association, which often detect but fail to replicate findings of main effect genes. While undoubtedly many of the original positive results are false-positives, true effects may not be replicated for many reasons, including population stratification and true differences in genetic etiology between study populations [20].

Current statistical approaches to detecting heterogeneity, such as the admixture test [21,22], are neither sensitive nor powerful and can merely account for, not resolve, any underlying heterogeneity. In addition, while a small number of supervised computational methods exist for discovering gene-gene interactions, the power of these methods drops dramatically when locus or trait heterogeneity is present [23,24]. Phenotypic data can be utilized to improve the performance of these methods in the face of locus or trait heterogeneity by facilitating heuristic stratification of data. However, for many diseases, little detailed phenotypic data has been collected consistently in combination with genotypic data. It is for these reasons that an unsupervised method, which does not rely on phenotypic data, is needed to mine potentially heterogeneous genotypic data as a means of data stratification and hypothesis generation.

For genetic factors involving heterogeneity, there are multiple independent (predictor) variables or else multiple dependent (outcome) variables that complicate the analysis by creating a heterogeneous model landscape. In the case of locus heterogeneity, multiple predictor variables (i.e., multiple loci) are present, some of which may be unmeasured or unobserved and, therefore, unavailable for inclusion in the disease model. In the case of trait heterogeneity, multiple outcome variables are present, which cannot or have not been distinguished based on the available phenotypic information. Gene-gene interactions create a rugged model landscape for statistical analysis. There is clear and convincing evidence that gene-gene interactions, whether synergistic or antagonistic, are not only possible but probably ubiquitous [18,19,25-28]. Thus, it is critical that complex genetic data sets be properly interrogated for possible underlying interactions.

### Statistical analysis

No one analytic method is superior in all respects for the range of complicating factors that might be present in a specific data set. Given the relative shortcomings of our current analyses in complex diseases, we need to greatly extend the range of available analytical tools. There is a critical need for extensive reevaluation of existing methodologies for complex diseases, as well as for massive efforts in new method development. It is important that empirical studies be conducted to compare and contrast the relative strengths and weaknesses of methods on specific types of problems. For example, while cluster analysis has shown promise in numerous scientific and mathematical fields, including genetics [29,30], its use with discrete genotypic data has not been adequately explored. Similarly, artificial neural networks modified with evolutionary computation have great potential for discovering nonlinear interactions among genes and environmental factors [31]. However, work is still ongoing to evaluate their limitations with regard to the heritability and effect sizes that can be detected.

Ultimately, though, the real power of existing and yet-to-be-developed methods lies in our ability to marry them into a comprehensive approach to genetic analysis, so that their relative strengths and weaknesses can be balanced and few alternative hypotheses are left uninvestigated. Because no single method adequately investigates heterogeneity and interaction issues simultaneously, we propose routinely taking a two-step approach to analysis. For example, clustering or ordered subset analysis [32] can be used first to uncover genotypic and/or phenotypic heterogeneity and to subdivide the data into more homogeneous groups. Then in a second step, specific tests of interactions, such as the S sum statistic approach [33,34,34] or the multifactor dimensionality reduction method [23,24,35-37] could be used to investigate gene-gene or gene-environment interactions within each of the homogenized subgroups. While this is not a perfect approach, it is an important improvement over the more common alternative of a single-pronged approach to analysis.

### Cluster analysis

For over 30 years, cluster analysis has been used as a method of data exploration [38]. Clustering is an unsupervised classification methodology, which attempts to uncover 'natural' clusters or partitions of data. It involves data encoding and choosing a similarity metric, which will be used in determining the relative 'goodness' of a clustering of data. No one clustering method has been shown universally effective when applied to the wide variety of structures present in multidimensional data sets. Instead, the choice of suitable methods is dependent on the type of target data to be analyzed. Clustering has been utilized widely for the analysis of gene expression (e.g., DNA microarray) data; however, its application to genotypic data has been limited [29].

Most traditional clustering algorithms use a similarity metric based on distance that may be inappropriate for categorical data such as genotypes. Newer methods have been developed with categorical data in mind and include extensions of traditional methods and application of probabilistic theory. Three such methods were chosen for comparison in the task of discovering trait heterogeneity using multilocus genotypes – Bayesian Classification [39], Hypergraph-Based Clustering [40], and Fuzzy *k*-Modes Clustering [41] – all of which are appropriate for categorical data.

## Results

### Descriptive statistics

**Figure 2:**
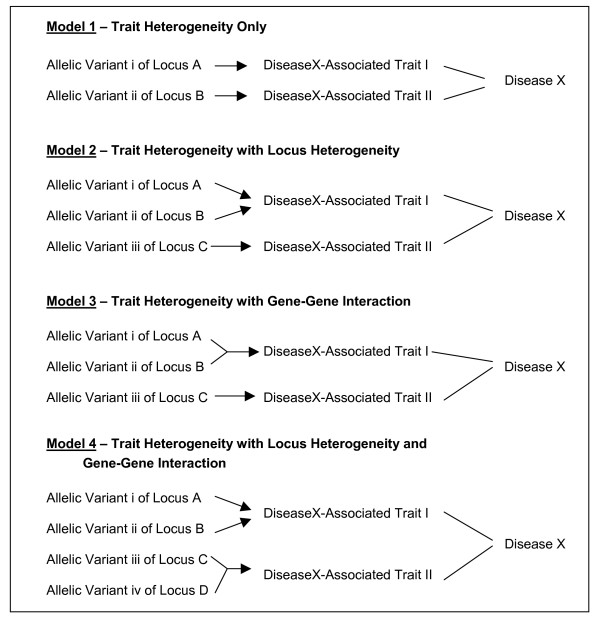
Structure of Genetic Models Used for Data Simulation

**Figure 3:**
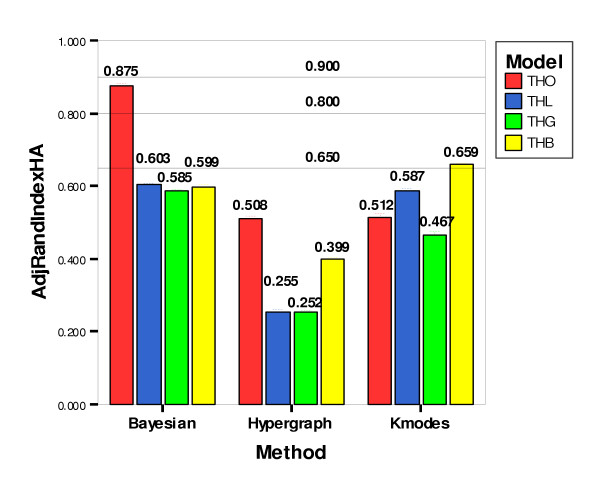
**Comparison of ARI_HA _Means by Method and Model**. Comparison of Hubert-Arabie Adjusted Rand Index means by method and model. Bars represent means, and error bars, which are very short and may be difficult to see, represent 95% confidence intervals. Horizontal lines represent thresholds for quality of cluster recovery: 0.90 for excellent recovery, 0.80 for good recovery and 0.65 for moderate recovery.

The Hubert-Arabie Adjusted Rand Index (ARI_HA_) was chosen as the standard for measuring clustering result fitness [42]. An ARI_HA _score of 0.90 indicates excellent cluster recovery, 0.80 good recovery, and 0.65 moderate recovery. Mean ARI_HA _values for Bayesian Classification, Hypergraph Clustering and Fuzzy *k*-Modes Clustering were 0.666, 0.354 and 0.556, respectively. Confidence intervals around the means were also produced to demonstrate the preciseness of the ARI_HA _measurements. The results for each method across all datasets are presented in Table 1. Mean ARI_HA _values differed by genetic model type (Figure 2), with higher scores achieved on Trait Heterogeneity Only (THO) datasets for the Bayesian Classification and Hypergraph Clustering methods (Figure 3).

### Comparison of clustering methods

**Figure 4:**
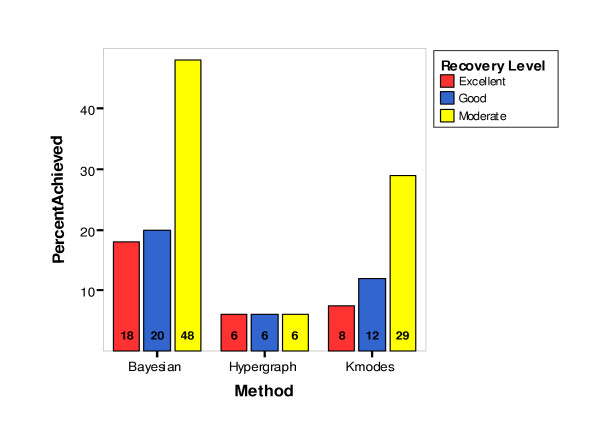
Percentage of Clustering Results Achieving Cluster Recovery Levels by Method

**Figure 5:**
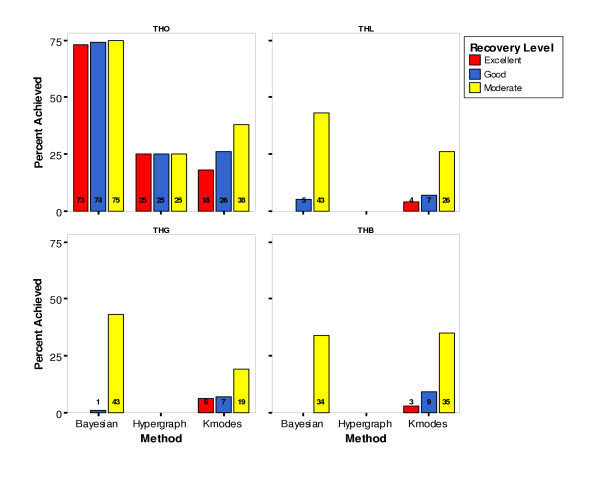
Percentage of Clustering Results Achieving Cluster Recovery Levels by Method and Model

Three categorical variables were constructed that could be tested using the nonparametric chi-square test of independence. The three variables were calculated as the number of clustering results achieving each of the three ARI_HA _cutoff values of 0.90 (for excellent cluster recovery), 0.80 (for good cluster recovery) and 0.65 (for moderate cluster recovery). Results are displayed as percentages by clustering method (Figure 4) and by clustering method and genetic model (Figure 5A). A chi-square test of independence was performed testing the null hypothesis that the number of clusterings achieving the specified ARI_HA _cutoff value was independent of the clustering method. The three methods performed significantly differently on each of the ARI_HA _cutoff statistics (Table 2). Bayesian Classification outperformed the other two methods. However, across all the dataset parameters, Bayesian Classification achieved moderate or better recovery on only 48% of the datasets (Figure 4).

The performance of the three clustering methods across different dataset parameters was evaluated in an attempt to find particular conditions under which one method consistently achieved good or excellent recovery (not just better recovery than the other two methods). For those datasets simulated under the THO model, Bayesian Classification performed well, with over 73 percent of its resulting clusterings achieving an ARI_HA _value of 0.90 or greater, indicating excellent recovery (Figure 5). For this subset of the datasets, Bayesian Classification outperformed the other two methods, and again there was a significant difference in performance across the three methods, as measured by a chi-square test of independence on each of the three new ARI_HA _cutoff statistics (Table 3). Analysis of the other simulation parameters failed to show as great a difference among methods where the 'winning' method performed as well as the Bayesian Classification performed in the THO datasets (data not shown). Thus, this subset of data was chosen for further investigation into the efficacy of using the Bayesian Classification method to uncover trait heterogeneity in **real **data.

### Applicability to real data

**Figure 6:**
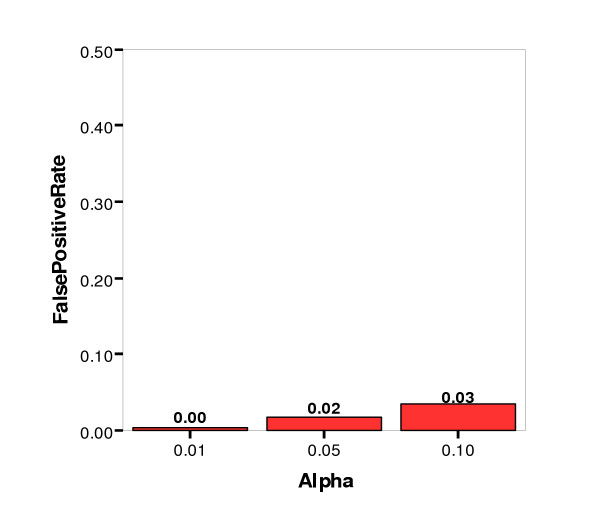
False Positive Rate by Significance Level (alpha)

**Figure 7:**
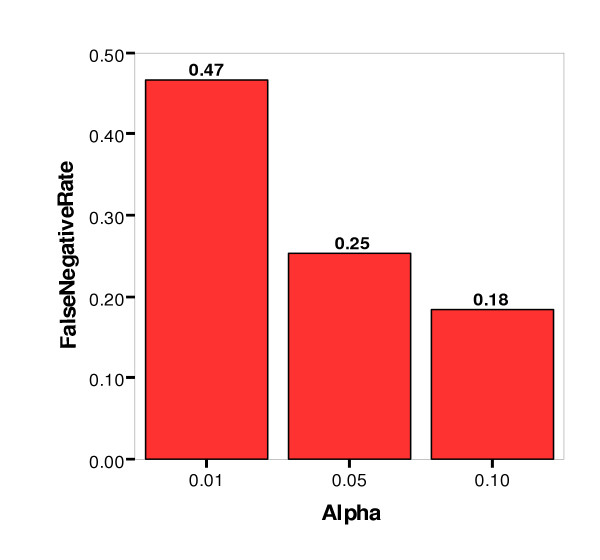
False Negative Rate by Significance Level (alpha)

To evaluate the validity of using the Bayesian Classification internal clustering metrics – class strength and cross-class entropy – as a proxy for the ARI_HA _(since ARI_HA _is unknown for real data), permutation testing was performed. Resulting p-values for ARI_HA_, average log of class strength and average cross class entropy were used to calculate false positive and false negative rates at three significance levels of 0.01, 0.05 and 0.10. A clustering result was considered a false positive if it was considered significant according to **either **average log of class strength or average cross class entropy but was not considered significant according to our ARI_HA _standard. A clustering result was considered a false negative if it was called not-significant according to **both **average log of class strength and average cross class entropy but was considered significant according to ARI_HA_. Figures 6 and 7 show the false positive and false negative rates, respectively, by alpha level.

**Figure 8:**
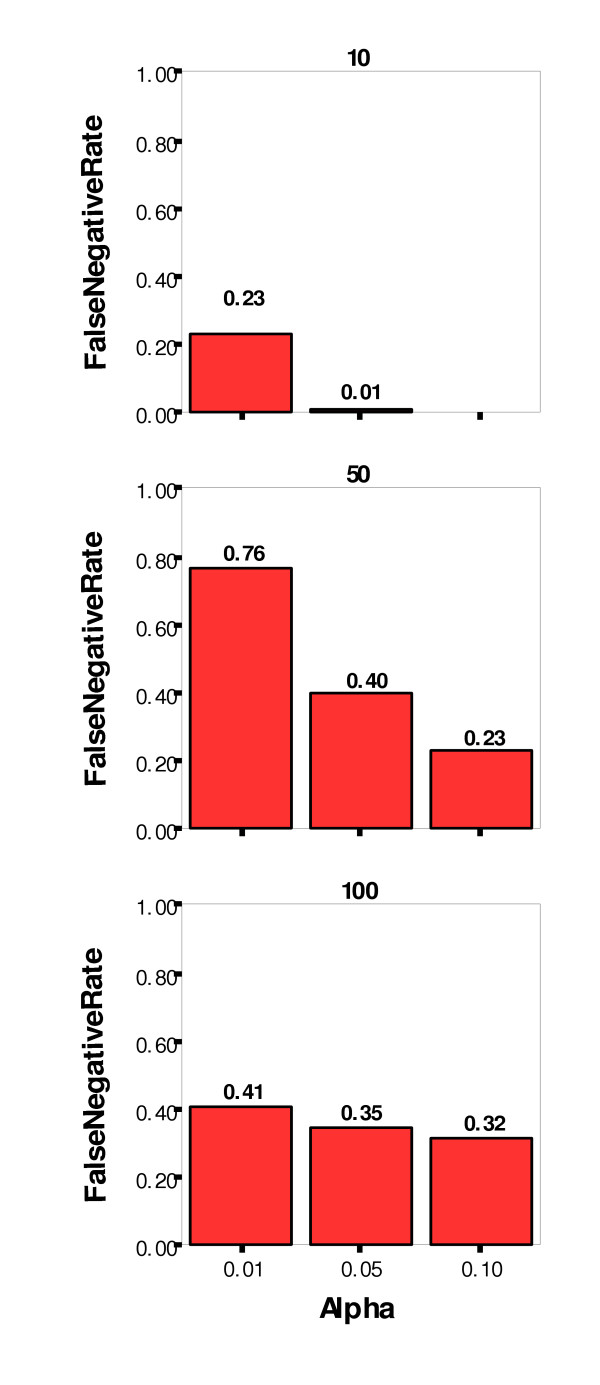
False Negative Rate by Significance Level (alpha) Paneled by Number of Nonfunctional Loci

**Figure 9:**
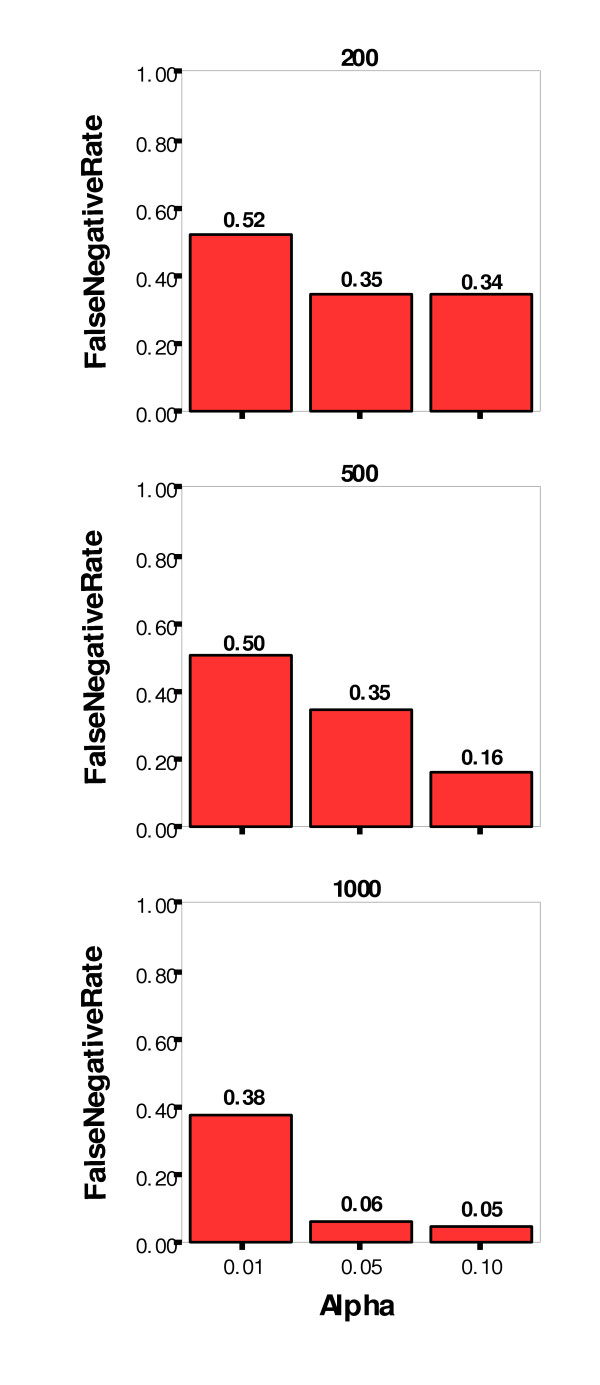
False Negative Rate by Significance Level (alpha) Paneled by Number of Affecteds

The false positive, or Type I, error rate was controlled very well at three percent or less for all three significance levels. The false negative, or Type II, error rate was not controlled as well, however. At the least stringent significance level (α = 0.10), the Type II error rate was 18 percent, and at the most stringent level (α = 0.01), the rate was 47 percent. Other simulation parameters were examined for their impact on the false negative rate, and Figures 8 and 9 show the false negative rate by alpha level paneled by number of nonfunctional loci and number of affecteds (sample size), respectively. As might be expected, the lowest false negative rates were achieved for datasets with the lowest number of nonfunctional loci (10) and the greatest sample size (1000).

## Discussion

### Data simulation

The new data simulation algorithm produced complex genotypic datasets that included trait heterogeneity, locus heterogeneity and gene-gene interactions. Most existing simulation software that attempt to simulate heterogeneity do so by allowing the user to specify what portion of the dataset is to be simulated under one model versus another, and the resulting individuals are simply combined into one dataset. In the new algorithm, however, the disease penetrance models, which were used to simulate the data, were constructed so that overall prevalence levels were controlled, allowing naturally occurring overlaps, in which some individuals would have both traits (and their associated multilocus genotypes) by chance. This novel data simulation algorithm should prove very useful for future studies of other proposed genetic analysis methods for complex diseases.

### Comparison of clustering methods

The Bayesian Classification method outperformed the other two methods across most dataset parameter combinations, with the exception of the most complex model (THB) on which Fuzzy *k*-Modes Clustering performed best. When the results were further examined to find a set of parameters for which one or more methods performed well, Bayesian Classification was found to have achieved excellent recovery for 73% of the datasets with the THO model and achieved moderate recovery for 56% of datasets with 500 or more affecteds and for 86% of datasets with 10 or fewer nonfunctional loci (data not shown here). Neither Hypergraph Clustering nor Fuzzy *k*-Modes Clustering achieved good or excellent cluster recovery even under a restricted set of conditions.

Bayesian Classification was obtained as closed-source software, for which there were numerous parameters, which could have been optimized. Initial parameter settings were chosen as recommended by the authors based on the type of data being analyzed. However, it is possible that alternative settings may have yielded better results. For example, for datasets with the more complex genetic models, greater numbers of nonfunctional loci and smaller sample sizes, the maximum number of classification trials and/or the maximum number of classification cycles per trial may need to be longer, and those parameters concerned with convergence rate and stopping criteria may need to be changed to delay convergence. If improvements in performance could be achieved with reasonable time and resource tradeoffs, such changes would certainly be desirable. The results of this simulation study are encouraging enough to warrant further investigation of this matter.

It was disappointing that Hypergraph Clustering did not perform very well under most conditions, despite its intuitive appeal as a method that would find frequently-occurring multilocus genotypic patterns. The Hypergraph Clustering method has been reported to work well with very large variable sets (on the order of thousands), which have complex patterns for which large numbers of clusters (10–20+) were relevant [43]. However, there has been no examination of the method's performance on smaller variable sets. Thus, it is possible that the restricted patterns present in our multilocus genotypic data were too simple and sparse and that the method is simply tuned to search for more complex patterns. Also, we were required to devise a translation of the resulting partitioning of genotypes into a clustering of individuals. We tested several such translations and implemented the best process out of several tested. Oftentimes, even when the method correctly chose the functional genotypes to be in different partitions, too many other nonfunctional genotypes were also chosen, which meant that the difference between an individual's likelihood of belonging to one cluster versus another was too small, making the choice of cluster assignment almost arbitrary.

The Fuzzy *k*-Modes Clustering method performed comparably to Bayesian Classification for the more complex datasets and was much less computationally intensive. It has been widely reported that the performance of *k*-means algorithms is highly variable depending on the method of seeding the initial cluster centroids [44]. While we used the recommended method of selecting individuals from the dataset to serve as the initial cluster modes, we perhaps could have achieved better results if we implemented an additional step to ensure that the initial centroids were substantially dissimilar to each other. This is supported by evidence that when the Fuzzy *k*-Modes Clustering resulted in only one cluster (effectively no partitioning of the data), the initial centroids were very similar and the method had converged early so that individuals had equal probability of belonging to any of the clusters. In such cases, the individual was arbitrarily assigned to the first cluster, thereby leading to all other clusters being empty.

As expected, the simpler the model, the better the performance of the three clustering algorithms, with the exception that the Hypergraph Clustering and Fuzzy *k*-Modes Clustering methods performed somewhat better on the THB (Trait Heterogeneity with Both locus heterogeneity and gene-gene interaction) datasets than they did on the THL (Trait Heterogeneity with Locus heterogeneity) and THG (Trait Heterogeneity with Gene-gene interaction) datasets. Likewise, in general, the fewer the nonfunctional loci and the larger the sample size, the better the performance was.

### Applicability to real data

To determine the efficacy of using the Bayesian Classification method on real data, the reliability of its internal clustering metrics at finding good clusterings was evaluated. Using the combination of the average log of class strength and the average cross class entropy to determine significance, the false positive rate was controlled very well, at three percent or less for all three significance levels. The false negative rate was acceptably low (18 percent) for the less stringent significance levels of 0.10. However, it was high (47 percent) for the most stringent significance level of 0.01. Thus, if a clustering of data were called significant according to permutation testing using either the average log of class strength or the average cross class entropy, we can be quite confident that the result were real. Typically geneticists prefer to accept a higher false positive rate to increase power; however, there is indeed a trade-off between these two types of error. Valuable time and resources can be spent on follow-up studies, and it can be very detrimental to pursue leads that do not have a good chance of yielding new information about the disease under study.

## Conclusion

The efficacy of three clustering methodologies at uncovering trait heterogeneity in genotypic data was investigated. One method, Bayesian Classification, was found to perform very well under certain conditions (THO model) and to outperform the other methods. Permutation testing confirmed that the method could be used on real data with excellent Type I error control and acceptable Type II error control. By controlling the false positive rate so well, Bayesian Classification offers a comfortable degree of certainty with regard to the hypotheses that it generates. This is true at least when the underlying data structure is similar to that simulated under the THO model. Further investigation of how different parameter settings may improve the performance of Bayesian Classification is planned.

## Methods

### Data simulation

To compare the performance of clustering methodologies in the task of uncovering trait heterogeneity in genotypic data, datasets were needed in which such heterogeneity was known to exist. Since there are no well-characterized real datasets available that fit this description, a simulation study was needed. Genetic models that contained two binary disease-associated traits, such that there is trait heterogeneity among 'affected' individuals, were used. In addition, some of the models incorporate locus heterogeneity, a gene-gene interaction, or both. Figure 2 depicts the structure of the four genetic models used to simulate the genotypic data.

Four prevalence levels were simulated for each genetic model: (1) fifteen percent, which is characteristic of a common disease phenotype such as obesity [45], (2) five percent, which is characteristic of a relatively common disease such as prostate cancer [46], (3) one percent, which is characteristic of a less common disease such as schizophrenia [47], and (4) one tenth of one percent, which is characteristic of a more uncommon disease such as multiple sclerosis [48]. Three realistic levels of sample size were simulated for each model: 200, 500 and 1000 affected individuals. Finally, four levels of non-functional loci were simulated: 0, 10, 50 and 100. The inclusion of non-functional loci adds a random noise effect that is present in real candidate gene studies in which the functional locus or loci are among many more suspected but actually non-functional loci. All loci, including the functional loci, were simulated to have equal biallelic frequencies of 0.5.

Although the above parameter settings are by no means exhaustive of the biologically plausible situations, the outlined conditions are reasonable and specify 192 different sets of data specifications due to the combinatorial nature of the study design. To have adequate power to detect a difference in performance among clustering methodologies, 100 datasets per set of parameters, resulting in a total of 19,200 simulated data sets, were simulated.

**Figure 10:**
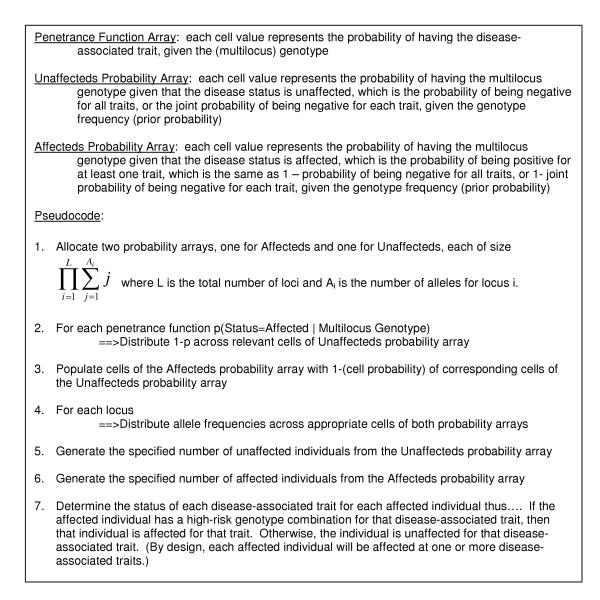
**Novel Data Simulation Algorithm**. Simulates trait heterogeneity, locus heterogeneity and gene-gene interactions in an epidemiologically-sound manner. The inputs are penetrance function arrays, which are translated into probability arrays for affecteds and unaffecteds, separately. Then affected and unaffected individuals (with multilocus genotypes) are simulated from those respective arrays.

For the purposes of simulating this data, a novel data simulation algorithm capable of incorporating these complex genetic factors in an epidemiologically-sound manner was designed and developed (Figure 10). Penetrance is the probability of having a particular trait given a specific genotype (single or multilocus). Prevalence, on the other hand, is the percentage of individuals in a population that have a particular trait. The penetrance levels of the two simulated disease-associated traits are constrained by the overall prevalence level of the simulated disease. The two traits were simulated to contribute equally to the prevalence of the associated disease (fifty percent trait heterogeneity), such that a small but naturally occurring degree of overlap would be present, representing individuals having both disease-associated traits, instead of just one or the other. These penetrance tables are inputs for the new data simulation algorithm.

**Figure 11:**
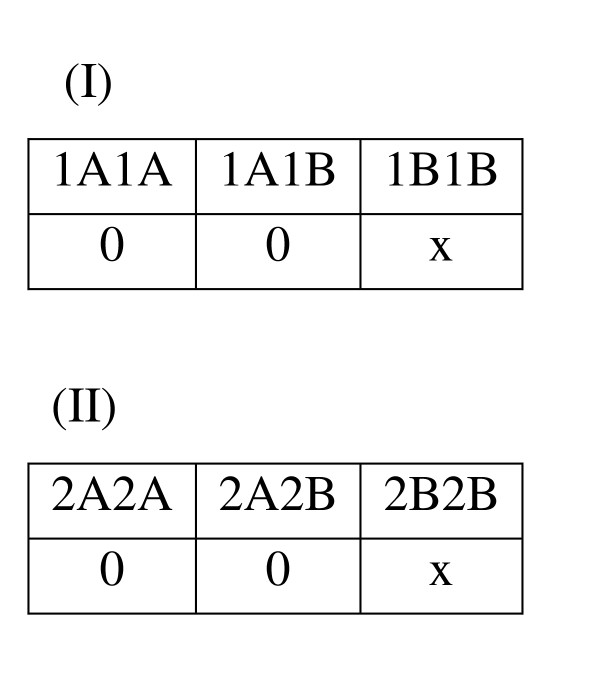
**Genetic Model THO (Trait Heterogeneity Only)**. The penetrance tables for Trait I and Trait II are presented. Cell values indicate penetrance level, or the probability of having the trait, given the corresponding multilocus genotype. For each of the two traits, a Mendelian recessive genetic model is used, in which the trait is penetrant only when two copies of the high risk (B) allele are present. The penetrance (x) is constrained by the desired overall disease prevalence to be simulated (0.001, 0.01, 0.05 or 0.15).

For one fourth of the models, trait heterogeneity only is involved (not locus heterogeneity or gene-gene interactions), and there is one genetic risk factor for each of the two traits. Each locus acts in a recessive manner, such that affected individuals have both copies of the high-risk allele at the disease-associated "functional" locus (Figure 11). A naturally occurring degree of overlap between the two traits can result, such that some affected individuals have the high-risk genotypes for both traits.

**Figure 12:**
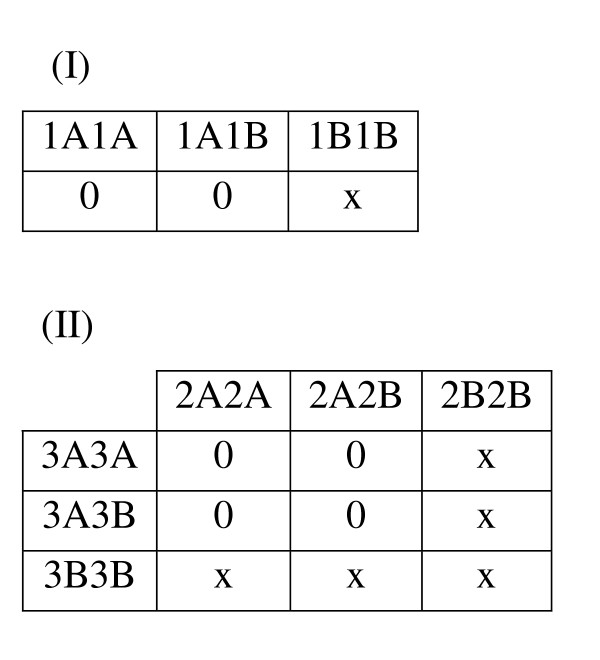
**Genetic Model THL (Trait Heterogeneity with Locus Heterogeneity)**. The penetrance tables for Trait I and Trait II are presented. Cell values indicate penetrance level, or the probability of having the trait, given the corresponding multilocus genotype. For Trait I, a Mendelian recessive genetic model is used, in which the trait is penetrant only when two copies of the high risk (B) allele are present. For Trait II, a locus heterogeneity model described by Li and Reich [49] is used, in which the trait is penetrant only when two copies of the high risk allele at one or both loci are present (in this case the B alleles for locus 2 and 3 are high risk).

In the second quarter of the datasets, alocus heterogeneity described by Li and Reich [49] was also simulated (Figure 12(II)) so that for one of the traits, there are two associated loci, each of which is responsible for roughly half of the individuals affected with the trait. In that locus heterogeneity model, each of the functional loci acts in a recessive manner, such that the disease-associated genotype for the locus consists of two copies of the high-risk allele. For the other trait, a recessive model was implemented, as described above (Figure 12(I)). By chance, there might be some affected individuals who have the high-risk genotype from the first trait as well as one of the high-risk genotypes from the second trait.

**Figure 13:**
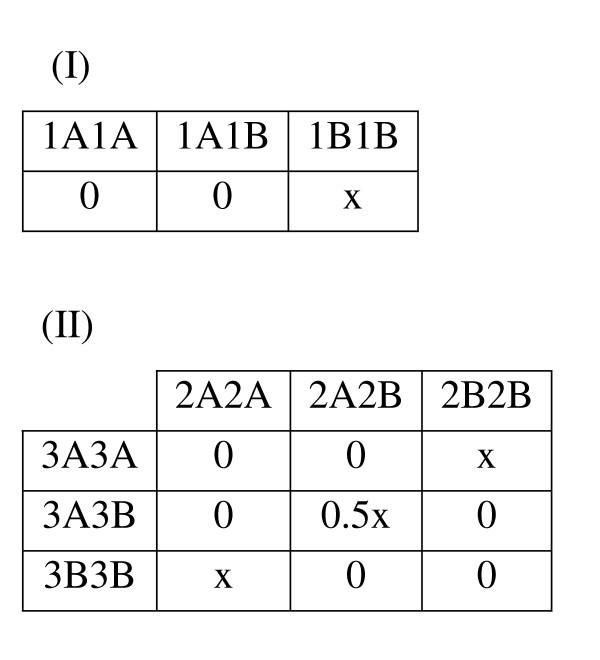
**Genetic Model THG (Trait Heterogeneity with Gene-Gene Interaction)**. The penetrance tables for Trait I and Trait II are presented. Cell values indicate penetrance level, or the probability of having the trait, given the corresponding multilocus genotype. For Trait I, a Mendelian recessive genetic model is used, in which the trait is penetrant only when two copies of the high risk (B) allele are present. For Trait II, the ''diagonal'' genetic model first described by Frankel & Schork [50] and later by Li and Reich [49] is used. Two loci (2 and 3) are involved, each with two alleles (A and B), and the trait is penetrant only when *exactly *two copies of the high risk allele from either locus are present.

In the third quarter of the datasets, a gene-gene interaction was simulated for one of the two traits. The "diagonal" gene-gene interaction model, first described by Frankel and Schork [50] and later by Li and Reich[49], which is nonlinear and nonadditive in nature, was used (Figure 13(II)). Under this model, a multilocus genotype is high-risk if it has exactly two high-risk alleles from either of the two associated loci. A multilocus genotype with fewer than or greater than two high-risk alleles is not associated with disease. For the other trait, a recessive model was implemented, as described above (Figure 13(I)). By chance, there might be some affected individuals who have the high-risk genotype from the first trait as well as one of the high-risk genotypes from the second trait.

**Figure 14:**
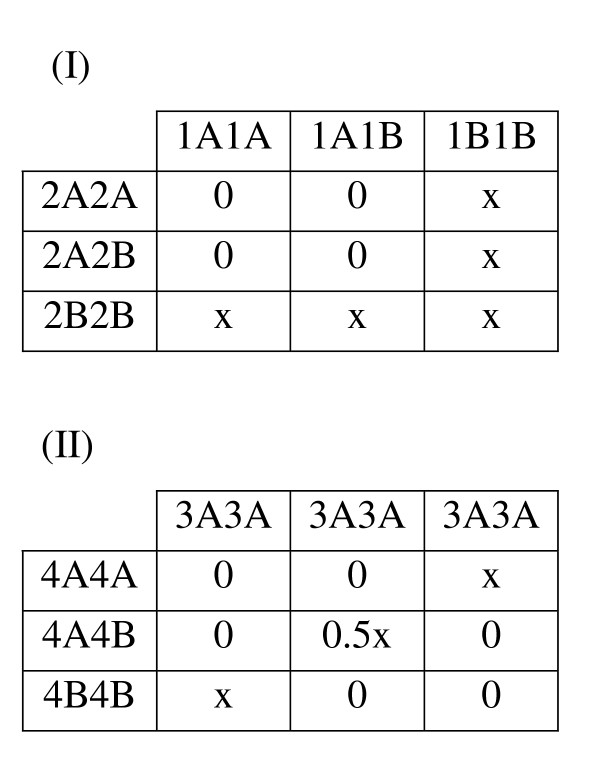
**Genetic Model THB (Trait Heterogeneity with Both Locus Heterogeneity and Gene-Gene Interaction)**. The penetrance tables for Trait I and Trait II are presented. Cell values indicate penetrance level, or the probability of having the trait, given the corresponding multilocus genotype. For Trait I, a locus heterogeneity model described by Li and Reich [49] is used, in which the trait is penetrant only when two copies of the high risk allele at one or both loci are present (in this case the B alleles for locus 2 and 3 are high risk). For Trait II, the ''diagonal'' genetic model first described by Frankel & Schork [50] and later by Li and Reich [49] is used. Two loci (2 and 3) are involved, each with two alleles (A and B), and the trait is penetrant only when *exactly *two copies of the high risk allele from either locus are present.

In the fourth quarter of the datasets, one trait is simulated to involve locus heterogeneity (Figure 14(I)), while the other is simulated to involve the "diagonal" gene-gene interaction, as described above (Figure 14(II)). Thus, there are some affected individuals who, by chance, will have one high-risk genotype from the first trait as well as one high-risk genotype from the second trait.

The input file for each of the clustering methods described below includes genotype and trait status information. Each row is a single individual. Column headings include unique individual number, trait status (affected for Trait 1, Trait 2, or both), and all simulated loci. Genotypes for each locus are encoded nominally (not ordinally), such that no genetic model assumptions are incorporated. Loci are numbered, and alleles are lettered. Thus, for a given locus '3' that has two alleles 'A' and 'B', the three possible genotypes are '3A3A', '3A3B', and '3B3B'. A different nomenclature could easily be used, however, since the methods simply treat each genotype as a character string for labeling purposes only and do not attribute any meaning or order to them.

### Clustering methods

There exists a very large number of clustering algorithms and even more implementations of those algorithms. The choice of which clustering methodology to use should be determined by the kind of data being clustered and the purpose of the clustering [51]. Genotypic data is categorical, which immediately narrows the field of appropriate methods for this study to only a few. Three different clustering methodologies were chosen that are suitable for categorical data and are appealing due to their speed or theoretical underpinnings.

**Figure 15:**
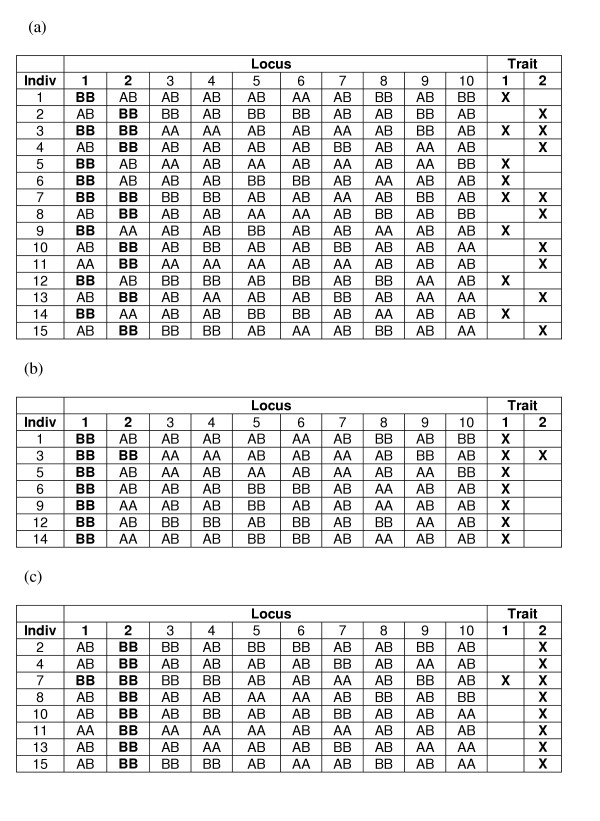
**Hypothetical Clustering of a THO Dataset**. (a) A small dataset that consistent Trait Heterogeneity Only (THO) genetic model (see Figure 11) is presented. All individuals with the high risk genotype (BB) at locus 1 have Trait I, and all individuals with the high risk genotype (BB) at locus 2 have Trait II. Some individuals have both high risk genotypes and, therefore, both traits. A successful clustering of this dataset might be one in which there are two clusters (b) and (c), such that one cluster contains only individuals who have Trait I (b) and the other cluster contains only individuals who have Trait II (c).

The goal of this clustering is to find a partitioning of the affected individuals based on multilocus genotypic combinations that maps onto the trait heterogeneity simulated in the data. For example, consider a dataset with 10 loci (numbered 1 to 10), each of which has two alleles (A and B), such that at each locus there are three possible genotypes (AA, AB and BB). It is likely that among affected individuals in the dataset, subsets of individuals will share specific genotypes or multilocus combinations of genotypes (such as 2B2B; or 3A3B and 9A9B together), either by chance or because such combinations are related to genetic background, phenotypic variability, or trait heterogeneity in some way. Thus, a successful clustering would be one in which all the individuals who were simulated to have Trait I end up in one or more clusters that do not have any individuals unaffected for Trait I and all individuals who were simulated to have Trait II end up in one or more distinct clusters that do not have any individuals unaffected for Trait II (Figure 15). (Those individuals, who by chance have both Trait I and Trait II, could be 'correctly' placed in any cluster.) Such a clustering would effectively eliminate the noise present among affected individuals due to trait heterogeneity. In the case where locus heterogeneity is also simulated, an even more successful clustering would be one in which there are two or more Trait II clusters, each of which has only those individuals who have a specific high-risk genotype (e.g., 2B2B from Figure 12) and none that do not.

The first clustering method is Bayesian Classification [39,52]. The corresponding AutoClass software is freely available from Peter Cheeseman at the NASA Ames Research Center. Bayesian Classification (BC) aims to find the most probable clustering of data given the data and the prior probabilities. In the case of genotypic data, prior probabilities are based on genotype frequencies, which for the purpose of the proposed data simulations are set in accordance with Hardy-Weinberg equilibrium and equal biallelic frequencies of 0.5. The most probable clustering of data is determined from two posterior probabilities. The first involves the probability that a particular individual belongs to its 'assigned' cluster, or otherwise stated as the probability of the individual's multilocus genotype, conditional on it belonging to that cluster, with its characteristic genotypes. The second posterior probability involves the probability of a cluster given its assigned individuals, or otherwise stated as the probability of the cluster's characteristic genotypes, conditional on the multilocus genotypes of the individuals assigned to that cluster.

In actuality, individuals are not 'assigned' to clusters in the hard classification sense but instead in the fuzzy sense they are temporarily assigned to the cluster to which they have the greatest probability of belonging. Thus, each individual has its own vector of probabilities of belonging to each of the clusters. The assignment of individuals is also not considered the most important result of the clustering method. A ranked listing is produced of all loci in the dataset with their corresponding normalized "attribute influence" values (ranging between 0 and 1), which provide a rough heuristic measure of relative influence of each locus in differentiating the classes from the overall data set. Thus, emphasis is placed on the identification of which attributes, or loci, are most important in producing the clustering. This information that can then be used to more directly stratify affected (and/or unaffected) individuals, for instance, by using the top n most influential loci identified, and to enable meaningful interpretation of the clustering result.

The second method is Hypergraph Clustering [40]. It has been implemented in the hMETIS software, which is freely available from George Karypis at the University of Minnesota. Hypergraph clustering seeks a partitioning of vertices, such that intracluster relatedness meets a specified threshold, while the weight of hyperedges cut by the partitioning is minimized. In this case, vertices represent single locus genotypes, hyperedges represent association rules, and hyperedge weights represent the strength of the association rules. For instance, if a specific genotype at one locus co-occurs with a specific genotype at another locus, an association rule linking those two genotypes would be created, and that rule would have a weight equivalent to the proportion of individuals in the dataset that had both of those genotypes. Thus, for our purposes, association rules are multilocus genotype combinations that are found in the dataset. The freely available LPminer program was used to generate the association rules [53]. LPminer searches the database for multilocus genotype combinations that appear together with substantial frequency (above a prespecified "support" percentage) and outputs this info as a list of association rules. hMETIS takes these association rules and uses them to create a hypergraph in which single locus genotypes are vertices and association rules dictate the presence and weight of hyperedges. hMETIS creates a partition of the hypergraph such that the weight of the removed hyperedges is minimized. It achieves this by using a series of phases, somewhat analogous to the stages of a simulated annealing algorithm, in an attempt to avoid making decisions which are only locally (not globally) optimal.

**Figure 16:**
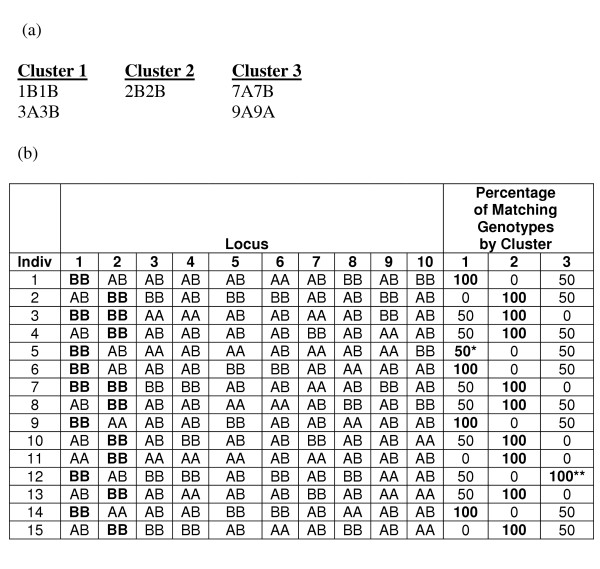
**Example of Post-processing of Hypergraph Clustering Result**. Hypergraph clustering produces a clustering of genotypes, instead of individuals. Thus, a clustering of individuals must be induced from this clustering of genotypes. As described in the text, an individual in assigned to the cluster for which it has the highest percentage of matching genotypes. Given the dataset presented in Figure 15(a) and a clustering of genotypes that is presented here (a), a clustering of individuals can be induced (b). For each individual (row), the percentage of matching genotypes that is highlighted indicates to which cluster the individual becomes assigned. Notice that for individual 5, there is a tie between the percentage of matching genotypes for clusters 1 and 3. In such cases, we arbitrarily assign the individual to the lower numbered cluster. Since cluster 3 does not contain any high-risk genotypes, it does not facilitate the goal of creating a clustering that maps to the simulated trait heterogeneity, and in the case of individual 12, it ends up capturing an individual who would preferably be clustered in cluster 1.

This process results in a partitioning (or clustering) of the genotypes in a dataset. If a single dataset was being analyzed, this information by itself could be sufficiently helpful since it would provide information about which multilocus genotypes appear with such frequency that they characterize groups of individuals. Individuals could be directly stratified using such multilocus combinations (similar to the way attribute influence values in the Bayesian Classification method could be used). However, for the purpose of comparing the results of Hypergraph Partitioning to those of the other two methods, which produce clusters, or partitions, of individuals (not genotypes), such a partitioning of individuals still needed to be created. Since a given individual could have more than one of the multilocus genotypes specified by different hyperedges in the final partitioning, the partitioning of individuals was not entirely straightforward. Thus, a heuristic was devised such that each individual would be assigned to the partition, or cluster, for which it had the highest percentage of matching genotypes (Figure 16). More specifically, for each cluster, the number of loci represented by one or more genotypes in that cluster was determined (L_c_). Then, for each individual, for each cluster, the number of matching genotypes between the cluster and the individual (M_ic_) was divided by L_c_, producing a vector of similarity percentages per individual, similar to the vector of probabilities used by the Bayesian Classification and Fuzzy *k*-Modes Clustering methods. Each individual was then assigned to the cluster with which it had the greatest similarity.

**Figure 17:**
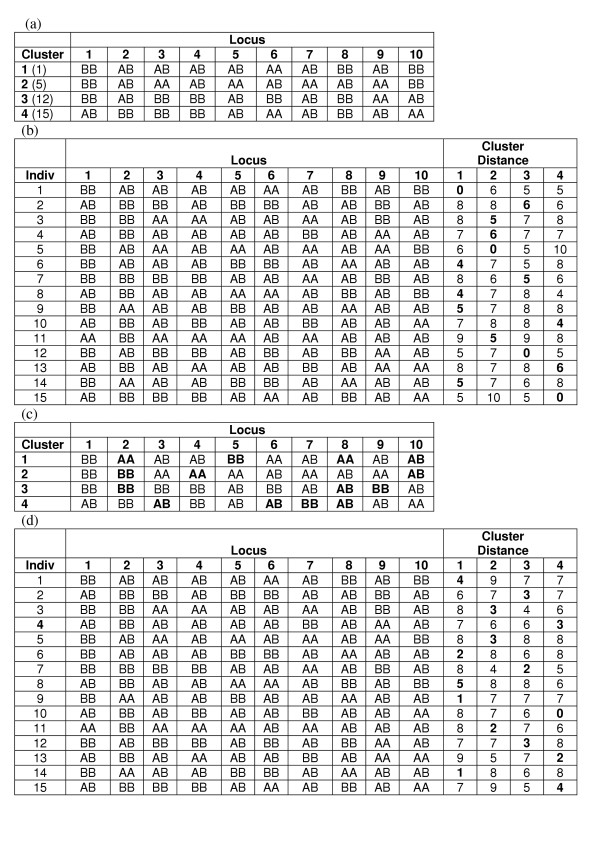
**Example of *k*-Modes Clustering**. In this example, the same dataset presented in Figure 15 is used to demonstrate the different steps involved the *k*-modes clustering algorithm. In this example, k was chosen to be 4, such that four clusters will initially be formed. (a) The cluster centroids are seeded by randomly selecting the genotypes of actual individuals in the dataset. The number in parentheses beside the cluster number is the individual used to seed that cluster. (b) Individuals are then compared to each of the cluster centroids, and the number of nonmatching genotypes between each cluster centroid and that individual are recorded. The individual is then assigned to the cluster for which it had the fewest number of nonmatching genotypes (in bold). (c) The next step is to update the cluster centroids based on the individuals now assigned to the clusters. The mode genotype among individuals assigned to a cluster becomes the centroid genotype at that locus. Genotypes that changed from the initialization to the update are shown in bold. (d) After the centroids are updated, the individuals are reevaluated as to which cluster they most closely resemble and are assigned to that cluster. Only cluster 4 was assigned to a different cluster than it was previously. Steps (c) and (d) are repeated until no genotypes are changed in any cluster centroid and no individuals' cluster assignments are changed.

The third clustering method is Fuzzy *k*-Modes Clustering [41]. *k*-Modes is a trivial extension of the popular *k*-means algorithm to categorical data. In both methods, cluster centroids can be initialized at random or by one of many seeding strategies [44], and individuals are assigned to their nearest cluster centroids. Then, cluster centroids are reevaluated based on their newly assigned individuals. For the *k*-means algorithm, the centroid is calculated as the mean vector of genotypes across individuals. However, for nominal data, such means are not necessarily meaningful, and the *k*-modes algorithm instead determines the centroid as the mode vector of genotypes across individuals. Genotypes are encoded nominally (not ordinally), such that no two genotypes are considered 'closer' than another two, and the 'distance' between an individual and a centroid is calculated as the cumulative number of non-matching genotypes across all loci. After cluster centroids are reevaluated, individuals are again assigned to their nearest centroids, and this process is repeated until the assignment of individuals to clusters does not change. Figure 17 demonstrates the first steps of the *k*-modes clustering, using the same dataset presented in Figures 15 and 16. The straightforward algorithm was developed in the C++ language. The number of clusters (*k*) was prespecified to be 2, 3, 4, 5 or 6. All five possible *k *were run for each dataset. Each cluster centroid was initially set to the values of a randomly selected individual in the dataset being analyzed. Both a 'fuzzy' and a 'hard' version of the *k*-modes algorithm were implemented and tested, and while their results on test datasets were comparable, the fuzzy version did perform slightly better and provided more information, which could be used for interpretation of results. Thus, the fuzzy version was chosen for use in these analyses.

### Statistical analysis

#### Comparison of clustering methods

Each clustering method has its own metric(s) for evaluating the "goodness" of a clustering of data. Since these methods are being tested on simulated data, classification error of a given clustering can be calculated as the number of misclassified individuals divided by the total number of individuals. However, simple classification error has its disadvantages. Firstly, in cases such as this where there is overlap between the known classes, the researcher must make an arbitrary decision as to when individuals who have been simulated to have both traits, not just one or the other, are considered to be misclassified. The decision about error is equally arbitrary when the number of resulting clusters is greater than the number of known classes. For instance, if the individuals belonging to one class were divided into two classes by the clustering algorithm, calculating classification error would require either (1) that none of those individuals be considered incorrectly classified, since they are all in homogenous clusters, or else (2) that all individuals from one of those clusters be considered misclassified. Neither choice seems to satisfactorily capture the "goodness" of the clustering result. Subsequently, it is not advisable to compare the classification error of two clustering results for which the number of clusters differs.

It is for these reasons alternative cluster recovery metrics were investigated. The Hubert-Arabie Adjusted Rand Index (ARI_HA_) addresses the concerns raised by classification error and was, therefore, chosen to evaluate the goodness of clustering results from the three clustering methods being compared [42]. Calculation of the ARI_HA _involves determining (1) whether pairs of individuals, who were simulated to have the same trait, are clustered together or apart and (2) whether pairs of individuals, who do not have the same trait, are clustered together or apart. The ARI_HA _is robust with regard to the number of individuals to cluster, the number of resulting clusters, and the relative size of those clusters [54]. It is, however, sensitive to the degree of class overlap, which is desirable since it will penalize more for good clusterings that occur by chance than classification error would. When interpreting ARI_HA _values, 0.90 and greater can be considered excellent recovery, 0.80 and greater is good recovery, 0.65 and greater reflects moderate recovery, and less than 0.65 indicates poor recovery. These values were derived from empirical studies showing observations cut at the 95th, 90th, 85th and 80th percentiles corresponded to ARI_HA _values of 0.86, 0.77, 0.67 and 0.60 respectively [54].

The ARI_HA _was used as the gold standard measure to compare the performance of the three clustering methods. Three categorical variables were created that could be tested using the nonparametric chi-square test of independence. The ARI_HA _values were discretized into a 1 or 0 depending on whether they met or exceeded the cutoff values for excellent, good and moderate cluster recovery, as described above. A chi-square test of independence was performed testing the null hypothesis that the number of clusterings achieving a certain ARI_HA _value was independent of the clustering method. Five percent was chosen as the significance level (alpha). An evaluation was performed of whether one method significantly outperformed the others and whether that method performed satisfactorily accordingly to the ARI_HA_.

#### Applicability to real data

As a reminder, the ultimate goal of this research is to find a clustering method that works well at uncovering trait heterogeneity in real genotypic data. Unlike for the current simulation study, for real data to which clusters individuals belong is not known *a priori*, otherwise the clustering would not be necessary. Indeed, it is the goal of clustering to uncover natural clusters or partitions of data using the method-specific "goodness" metric as a guide. In preparation for application of a clustering method to real data, after choosing the superior method, that method's internal clustering metrics were analyzed using permutation testing to determine how good a proxy they are for ARI_HA_.

The Bayesian Classification method produces two internal clustering metrics for each resulting cluster, or class: (1) class strength, and (2) cross-class entropy. Class strength is a heuristic measure of how strongly each class predicts "its" instances and is reported as the log of class strength. Cross-class entropy is a measure of how strongly the class probability distribution function differs from that of the dataset as a whole. Because each metric is reported per resulting cluster, or class, the average metric value across clusters was calculated and utilized for evaluating cluster fitness.

The ratio of one hundred permuted datasets per simulated dataset was chosen, which should result in a reasonable approximation of the null distribution but would not put unreasonable strain on resources and time [55]. Genotypes were permuted within loci across individuals, such that the overall frequency of genotypes at any one locus was unchanged, but the frequency of multilocus genotypes was altered at random. This created a null sample in which the frequency of multilocus genotypes was no longer associated with trait status except by chance. The empirically-determined superior clustering method was applied to each permuted data set and both the internal clustering metric values and the ARI_HA _were determined. For each set of 100 permuted data sets, the significance of each of the simulated dataset results was determined based on whether they exceeded the values at the significance level in the corresponding null distribution. Ten percent was chosen as the acceptable Type I error rate since these methods serve as a means of data exploration to be followed by more rigorous, supervised analyses on individual clusters of the data. However, the more conventional levels of 0.05 and 0.01 were also evaluated.

Finally, the ability of permutation testing to preserve an acceptable Type I (false positive) error rate was evaluated at the three specified significance levels. A false positive was defined as a clustering result which had a p-value according to at either of the internal clustering metrics that was significant but had a p-value according to ARI_HA _that was not significant. The Type II (false negative) error rate was evaluated at the same alpha levels to determine the sensitivity for detecting trait heterogeneity when it is present. A false negative was defined as a clustering result which had a p-value that was not significant according to both of the internal clustering metrics but had a p-value that was significant according to ARI_HA_.

## Abbrevations

ARI_HA _Hubert-Arabie Adjusted Rand Index

THO Trait Heterogeneity Only

THL Trait Heterogeneity with Locus Heterogeneity

THG Trait Heterogeneity with Gene-Gene Interaction

THB Trait Heterogeneity with Both Locus Heterogeneity and Gene-Gene Interaction

## Authors' contributions

TATW conceived of the study, performed all data simulations, implemented the three clustering methods, conducted the comparative data analysis, and drafted the manuscript. JHM and JLH participated in the design of the study and helped to draft the background section of the manuscript. All authors read and approved the final manuscript.

## Figures and Tables

**Table 1:** Confidence intervals around ARI_HA _means by method.

			95% Confidence Interval	
**Method**	**Mean**	**Standard Error**	**Lower End**	**Upper End**
Bayesian	0.666	0.001	0.664	0.667
Hypergraph	0.354	0.001	0.352	0.355
Fuzzy *k*-Modes	0.556	0.001	0.555	0.558

**Table 2:** Overall results of Chi-Square Test of Independence. Overall results of chi-square test of independence testing the null hypothesis that the percentage of clustering results achieving the specified cluster recovery level does not differ across clustering methods.

**Cluster Recovery Statistic**	**χ^2^**	**df**	**p**
%Results achieving Excellent cluster recovery (ARI_HA _≥ 0.90)	1787	2	< 0.001
%Results achieving Good cluster recovery (ARI_HA _≥ 0.80)	1614	2	< 0.001
%Results achieving Moderate cluster recovery (ARI_HA _≥ 0.65)	8565	2	< 0.001

**Table 3:** Results of Chi-Square Test of Independence for Trait Heterogeneity Only datasets. Results of chi-square test of independence for Trait Heterogeneity Only datasets, testing the null hypothesis that the percentage of clustering results achieving the specified cluster recovery level does not differ across clustering methods.

**Cluster Recovery Statistic**	**Model**	**χ^2^**	**df**	**p**
%Results achieving Excellent cluster recovery (ARI_HA _≥ 0.90)	THO	3713	2	< 0.001
%Results achieving Good cluster recovery (ARI_HA _≥ 0.80)	THO	3107	2	< 0.001
%Results achieving Moderate cluster recovery (ARI_HA _≥ 0.65)	THO	2609	2	< 0.001
